# Differential cardiovascular risk profiles by sex among adults with CKD: a NHANES-based analysis

**DOI:** 10.3389/fcvm.2025.1544590

**Published:** 2025-05-02

**Authors:** Hannah T. Belikoff, Ramya Walsan, Cara M. Hildreth, Jacqueline K. Phillips

**Affiliations:** ^1^ Faculty of Medicine, Health and Human Sciences, Macquarie University, Sydney, NSW, Australia; ^2^ Australian Institute of Health Innovation, Macquarie University, Sydney, NSW, Australia

**Keywords:** chronic kidney disease, cardiovascular risk factors, sex-differences, NHANES, hypertension, anaemia, diabetes

## Abstract

**Introduction:**

Chronic kidney disease (CKD) and cardiovascular disease are closely interconnected, with cardiovascular disease the leading cause of death for those with CKD. This increased risk for those with CKD is partly attributed to shared risk factors between the conditions. These risk factors differ in presentation between females and males; however, further research is needed to better understand how sex influences cardiovascular risk factors among individuals with CKD.

**Methods:**

Data from the National Health and Nutrition Examination Survey (NHANES) from 2007 to 2018 was utilised. CKD was classified as an albumin-to-creatinine ratio ≥30 mg/g or an estimated glomerular filtration rate (eGFR) <60 ml/min/1.73 m^2^. Participants were categorized into GFR categories based on their eGFR results for further analysis. Two-way ANOVAs compared means across groups, and a Tukey's *post hoc* test was performed to assess the statistical significance between group means. Multivariable logistic regression was used to determine the association between cardiovascular risk factors and sex among individuals with and without CKD.

**Results:**

A total of 30,804 participants aged 20 years and older were included, in which 5,528 were classified as having CKD. Our analyses of participants by sex and GFR categories revealed that for both females and males, as renal function declined, systolic blood pressure increased while haemoglobin and haematocrit levels decreased. Multivariate logistic regression revealed that females with CKD demonstrated reduced odds for diabetes (OR: 0.53, CI: 0.42–0.66), hypertension (OR: 0.80, CI: 0.66–0.97), low haematocrit (OR: 0.47, CI: 0.40–0.56), and elevated triglycerides (OR: 0.75, CI: 0.63–0.88), yet exhibited increased odds of a high waist circumference (OR: 1.69, CI: 1.40–2.04) and low high-density lipoprotein cholesterol (HDL-C) (OR: 1.18, CI: 1.00–1.39), compared to males with CKD.

**Conclusion:**

Sex-based differences in cardiovascular risk factors among individuals with CKD reveal that females have lower odds of diabetes, hypertension, low haematocrit and elevated triglycerides, but higher odds of increased waist circumference and low HDL-C compared to males. These findings highlight the need to incorporate sex-specific perspectives into CKD research and management to improve personalized care.

## 1 Introduction

Chronic kidney disease (CKD), affecting over 850 million individuals globally (1), is closely associated with cardiovascular disease, with individuals facing a significantly elevated risk of cardiovascular events and mortality, particularly in advanced CKD stages (2). This heightened risk arises from both shared traditional risk factors, such as hypertension and diabetes (3), and CKD-specific mechanisms, including uremic toxin accumulation, disturbances in mineral metabolism and inflammatory effects heightened with CKD (4, 5). This overlap substantially increases cardiovascular risk for individuals with CKD, as evidenced by the 2024 United States Renal Data System (USRDS) report, which documented that over 50% of known-cause deaths in dialysis patients were attributed to cardiovascular disease (6), a pattern similarly observed in Australian and New Zealand populations (7). Furthermore, cardiovascular mortality risk increases as kidney function declines, underscoring the complex relationship between CKD and cardiovascular disease (8, 9).

An emerging area of focus is the interplay between CKD and cardiovascular disease and how their etiologies and outcomes differ by sex. While females generally have a lower risk of cardiovascular disease in the general population, this protective effect appears to diminish in the context of CKD (10, 11). For instance, our prior systematic review and meta-analysis demonstrated that females and males with CKD have comparable risks of cardiovascular mortality, challenging conventional assumptions about sex-based differences in cardiovascular health (12). In alignment with these findings, an Australia and New Zealand study evaluating sex differences in mortality among individuals with kidney failure found few differences between the sexes in overall mortality. However, when compared to the general population, female patients with kidney failure exhibited significantly greater excess mortality than their male counterparts, with this disparity most pronounced in deaths caused by cardiovascular disease (13). Sex differences in CKD may be explained by differences in how both traditional and CKD-specific cardiovascular risk factors interact with hormonal differences, socio-economic status, and metabolic responses such as disorders of mineral and bone metabolism, ultimately influencing susceptibility to disease and treatment outcomes (4, 11).

Understanding these sex-based differences is critical for improving the management of CKD-related cardiovascular complications. Despite growing recognition of sex-specific factors in cardiovascular disease risk, it remains unclear how these factors interact with declining renal function or whether they influence treatment efficacy differently in females vs. males. For example, how sex modifies cardiovascular risk factors and their association with declining glomerular filtration rate (GFR) is limited (4). Addressing these gaps is essential for developing tailored prevention and treatment strategies to optimize cardiovascular outcomes for both sexes.

The primary objective of this study is to examine the influence of sex on cardiovascular risk factors, both traditional and CKD-specific, in those with and without CKD, using cross-sectional data from the National Health and Nutrition Examination Survey (NHANES) spanning 2007–2018. Additionally, this study examined how cardiovascular risk factors change with declining renal function in each sex.

## 2 Materials and methods

### 2.1 Study population

The NHANES is described in full elsewhere (14). Briefly, it is conducted by the National Center for Health Statistics to assess the nutritional and health status of the United States population, by using a complex, multistage sampling design. The NHANES is a cross-sectional survey with a new cohort of participants in each two-year survey cycle in which it collects data from surveys, physical examinations, and laboratory assessments. All the participants sign an informed consent form. Data for this study were drawn from six NHANES two-year survey cycles from 2007 to 2018. Cardiovascular mortality data were extracted from the NHANES-linked National Death Index file (15), and linked to the NHANES two year survey cycles. Participants were excluded from the study if they were under the age of 20 or had missing serum creatinine, urinary albumin and creatinine values. After excluding these individuals (*n* = 29,045), the final sample size for the study was 30,804 participants.

### 2.2 Assessment of CKD

CKD was identified using albumin-to-creatinine ratio (ACR) or estimated glomerular filtration rate (eGFR) (16). eGFR was calculated using the CKD Epidemiology Collaboration equation, which estimates GFR using serum creatinine, age and sex of an individual (17). CKD was classified as an ACR ≥30 mg/g or an eGFR <60 ml/min/1.73 m^2^ (16). Participants were categorized into different GFR categories as per the KDIGO recommendations for further analysis (16).

### 2.3 Assessment of risk factors

In the NHANES, interview questions, physical examination and laboratory values were assessed at baseline, per standard NHANES protocol (18). Participants were classified as female or male based on their self-reported responses in the survey. Blood pressure was measured at a mobile examination center with three readings taken after five minutes of seating. The average of the three readings was used to determine systolic and diastolic pressure. Hypertension was defined as systolic blood pressure >140 mmHg, diastolic blood pressure >90 mmHg (19), or self-reported use of antihypertensive medication. Diabetes was identified through self-report. Body mass index (BMI) and waist circumference data were collected in the mobile examination center. Obesity was classified as a BMI of ≥30 kg/m^2^ (20). High waist circumference was classified as ≥88 cm in females and ≥102 cm in males (21).

Based on the laboratory findings data, elevated triglycerides were classified as ≥150 mg/dl (22), low haemoglobin as <12 g/dl in females or <13 g/dl in males (23), and low haematocrit as <36% for females and <41% for males (24). Low high density lipoprotein-cholesterol (HDL-C) was defined as <50 mg/dl for females and <40 mg/dl for males (25), and hypoalbuminemia was classified as <3.5 g/dl (26).

### 2.4 Covariates

Age was categorized into three groups: young ages (20–39 years), middle age (40–64 years), or older age (65 years and above) as per previous NHANES studies (27, 28). As per the classifications provided by the NHANES, ethnicity was characterized as white, Mexican American, other Hispanic, African American, or other. Level of education was categorized as less than 9th grade, 9th−11th grade, high school graduate, some college or college graduate and above. Marital status was characterized as married, widowed, divorced, separated, never married, or living with partner. Annual household income was categorized within the brackets of <$US20,000, $US20,000-$US34,999, $US35,000-$US54,999, $US55,000-$US74,999, $US75,000-$US99,999, and above $US100,000. For physical activity, we multiplied the frequency and duration of self-reported recreational vigorous exercise, recreational moderate exercise, work vigorous exercise, work recreational exercise and exercise for transportation. From this, we calculated the number of minutes of physical activity per week by summing up the total amount of reported exercise. Physical activity was characterized as adequate if individuals reported ≥150 min of moderate and/or vigorous exercise per week, as per the World Health Organization 2020 guidelines (29). A history of smoking was identified if participants answered “Yes” to both survey questions: “Have you smoked at least 100 cigarettes in your lifetime?” and “Do you currently smoke cigarettes?”.

### 2.5 Statistical analysis

Statistical analysis was performed using R version 4.2.2 (30). Appropriate survey weights provided by the NHANES were applied to account for non-response bias, as well as the complex survey design (14). Descriptive statistics described the study sample, including chi-square tests of independence and *t*-tests to compare socio-demographic characteristics in individuals with and without CKD, including for cardiovascular mortality. The mean [± standard deviation (SD)] of each risk factor was graphed by GFR category and sex, with two-way analysis of variance (ANOVA) tests used to compare means across groups. A Tukey range test for *post hoc* analysis was performed to identify which specific group means were significantly different from one another, as indicated by the ANOVA results.

Multivariable logistic regression analysis was used to examine the association between binary cardiovascular risk factors [diabetes (Y/N), hypertension (Y/N), obesity (Y/N), high waist circumference (Y/N), elevated triglycerides (Y/N), low haemoglobin (Y/N), low haematocrit (Y/N), low HDL-C (Y/N), and hypoalbuminemia (Y/N)] and sex. Models were run separately for individuals with and without CKD and were adjusted for age, ethnicity, level of education, household income level, marital status, physical activity level and smoking status. Adjusted odds ratios (ORs) and 95% confidence intervals (CIs) were estimated to determine the strength of association between sex and the cardiovascular risk factors in individuals with and without CKD. Statistical significance was set at *p* ≤ 0.05.

## 3 Results

### 3.1 Descriptive statistics

The study sample consisted of 30,804 participants, of which 5,528 (17.9%) individuals had CKD. The mean age of individuals with CKD was 62.9 ± 16.3 years and the mean age for those without CKD was 46.7 ± 16.5 years (*p* < 0.05). There were 410 (7.4%) cardiovascular deaths among those with CKD and 265 (1.1%) among those without CKD (*p* < 0.05). Individuals identifying as white comprised the largest group, accounting for 46.1% of individuals with CKD and 40.0% of individuals without CKD (Table 1). African Americans represented the second largest ethnic group, constituting 22.1% of individuals with CKD and 20.2% of those without. The diagnosis of diabetes was reported more frequently among individuals with CKD (31.3%) than those without it (9.1%) (Table 2). The prevalence of hypertension was markedly higher in individuals with CKD (71.3%) compared to individuals without CKD (33.1%). Individuals with CKD had a higher occurrence of low haemoglobin (20.5% vs. 8.1%), low haematocrit (29.9% vs. 14.7%), elevated triglycerides (43.9% vs. 25.1%), and hypoalbuminemia (3.6% vs. 1.7%), compared to individuals without CKD.

**Table 1:** Demographic characteristics of the study sample, NHANES (2007–2018).

Variables	Individuals with CKD		Individuals without CKD		χ^2^ (*p*-value)
	Number	%	Number	%	
Sex					
Female	2,630	52.4	12,953	51.2	0.12
Male	2,898	47.6	12,323	48.8	
Age categories					
20–39	653	11.8	9,603	38.0	<0.05
40–64	1,850	33.5	11,459	45.3	
65+	3,025	54.7	4,214	16.7	
Ethnicity					
Mexican American	735	13.3	4,002	15.8	<0.05
Other Hispanic	492	8.9	2,785	11.0	
White	2,550	46.1	10,115	40.0	
African American	1,219	22.1	5,101	20.2	
Other race	532	9.6	3,273	12.9	
Education					
Less than 9th grade	853	15.4	2,424	9.6	<0.05
9–11th grade	879	15.9	3,411	13.5	
High school graduate	1,329	24.0	5,656	22.4	
Some college	1,508	27.3	7,598	30.1	
College graduate or above	950	17.2	6,169	24.4	
Marital status					
Married	2,724	49.3	13,178	52.1	<0.05
Widowed	1,027	18.6	1,345	5.3	
Divorced	709	12.8	2,658	10.5	
Separated	197	3.6	863	3.4	
Never married	592	10.7	4,992	19.7	
Living with partner	271	4.9	2,232	8.8	
Annual income					
<$20,000	1,514	27.9	4,710	18.6	<0.05
$20,000–$34,999	1,169	21.1	4,545	18.0	
$35,000–$54,999	958	17.3	4,229	16.7	
$55,000–$74,999	490	8.9	2,731	10.8	
$75,000–$99,999	350	6.3	2,380	9.4	
$100,000+	533	9.6	4,322	17.1	
Physical activity					
Inadequate	3,102	56.1	9,730	38.5	<0.05
Adequate	2,426	43.9	15,546	61.5	
History of smoking					
No	2,788	50.4	14,447	57.2	<0.05
Yes	2,740	49.6	10,829	42.8	

**Table 2:** Cardiovascular risk factor prevalence for individuals with and without CKD in the sample, NHANES (2007–2018).

Variables	Individuals with CKD		Individuals without CKD		χ^2^ (*p*-value)
	Yes % (*n*)	No % (*n*)	Yes % (*n*)	No % (*n*)	
Diabetes	31.3 (1,732)	68.7 (3,796)	9.1 (2,292)	90.9 (22,984)	<0.05
Hypertension	71.3 (3,876)	28.7 (1,562)	33.1 (8,133)	66.9 (16,464)	<0.05
Obesity	46.1 (2,548)	53.9 (2,980)	37.5 (9,480)	62.5 (15,796)	<0.05
Low HDL-C	36.6 (2,021)	63.4 (3,507)	30.6 (7,740)	69.4 (17,528)	<0.05
High waist circumference	69.0 (3,528)	31.0 (1,583)	55.1 (13,431)	44.9 (10,965)	<0.05
Low haemoglobin	20.5 (1,130)	79.5 (4,385)	8.1 (2,048)	91.9 (23,175)	<0.05
Low haematocrit	29.9 (1,647)	70.1 (3,868)	14.7 (3,697)	85.3 (21,526)	<0.05
Elevated triglycerides	43.9 (2,427)	56.1 (3,098)	35.1 (8,864)	64.9 (16,393)	<0.05
Low albumin	3.6 (197)	96.4 (5,331)	1.7 (418)	98.3 (24,858)	<0.05

*p*-value indicates the statistical differences between those with and without CKD.

### 3.2 Analysis of risk factors by sex and GFR category

**Figure 1:**
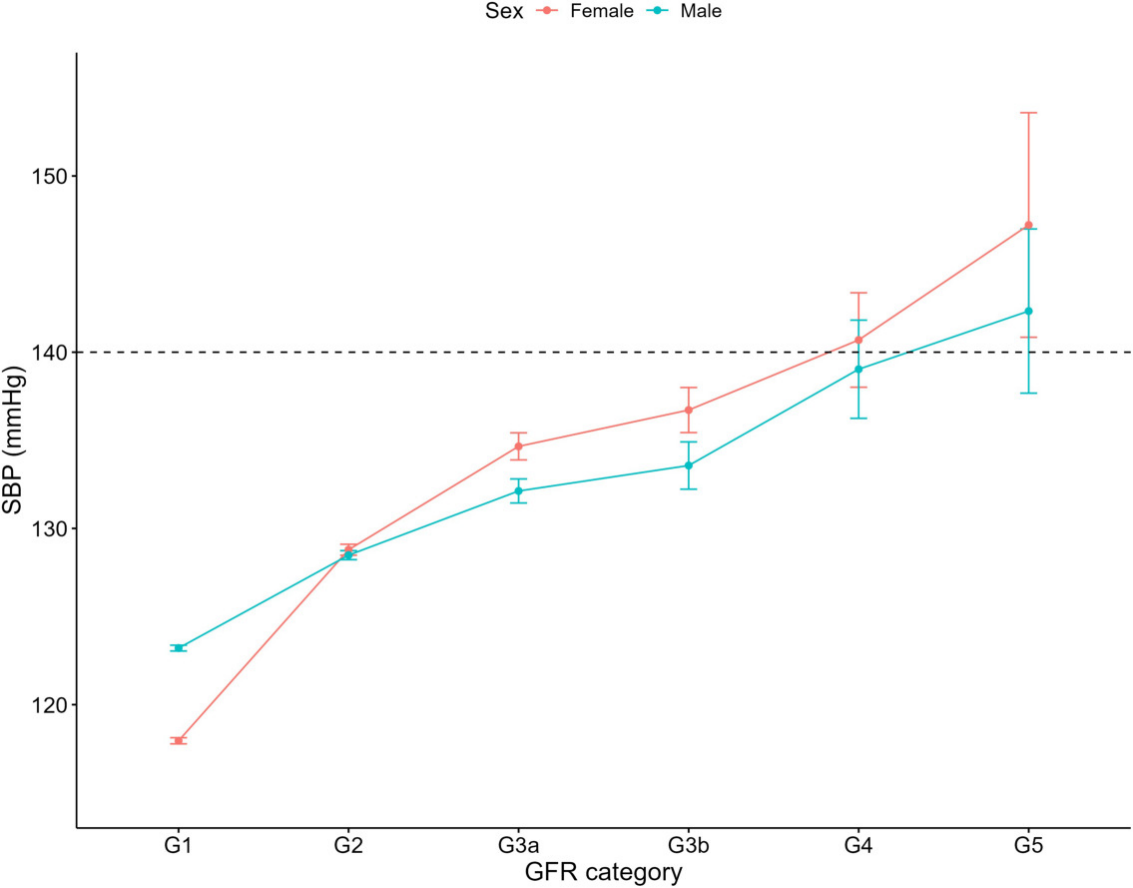
Systolic blood pressure within each sex by GFR categories, NHANES (2007–2018). Figure illustrates data for systolic blood pressure (SBP; mmHg) for females and males, grouped by GFR category. Dashed line illustrates threshold of 140 mmHg which was used to define if an individual was hypertensive.

**Figure 2:**
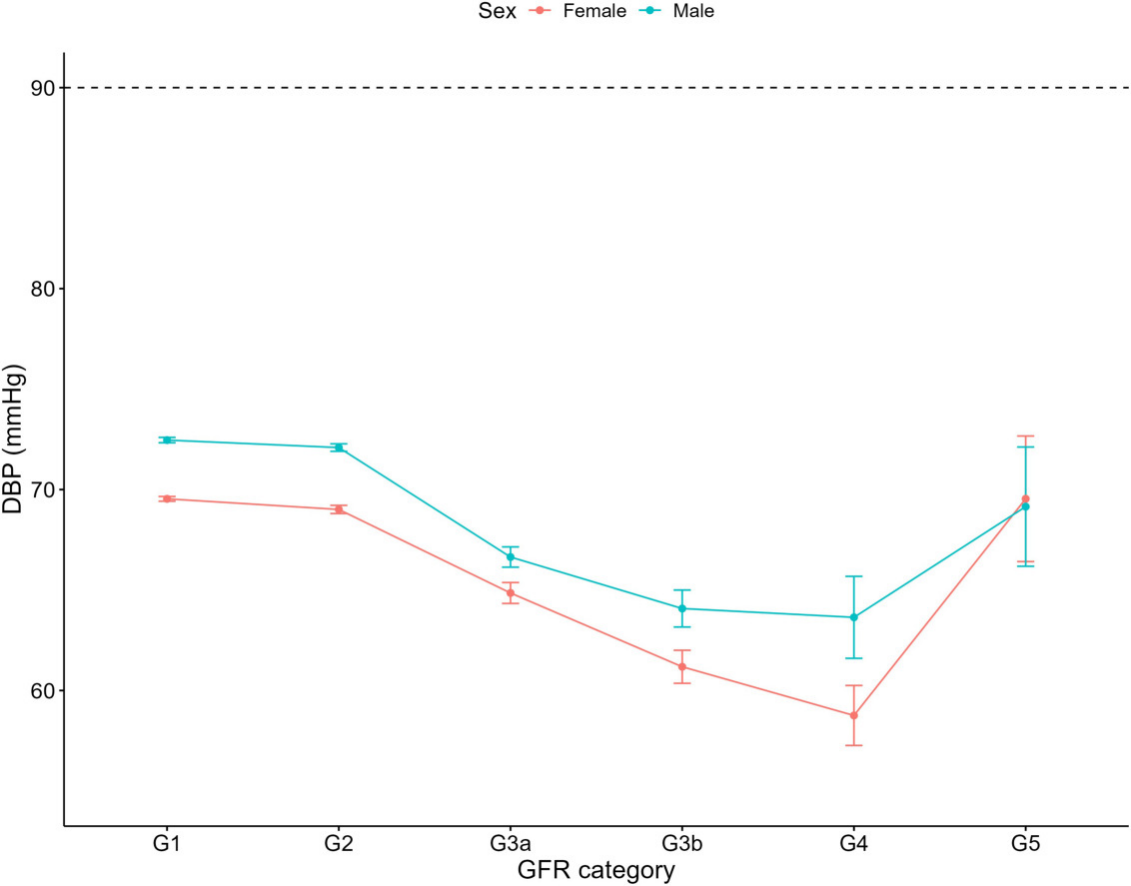
Diastolic blood pressure within each sex by GFR category, NHANES (2007–2018). Figure illustrates diastolic blood pressure (DBP; mmHg) for females and males, grouped by GFR category. Dashed line illustrates threshold of 90 mmHg which was used to define if an individual was hypertensive.

**Figure 3:**
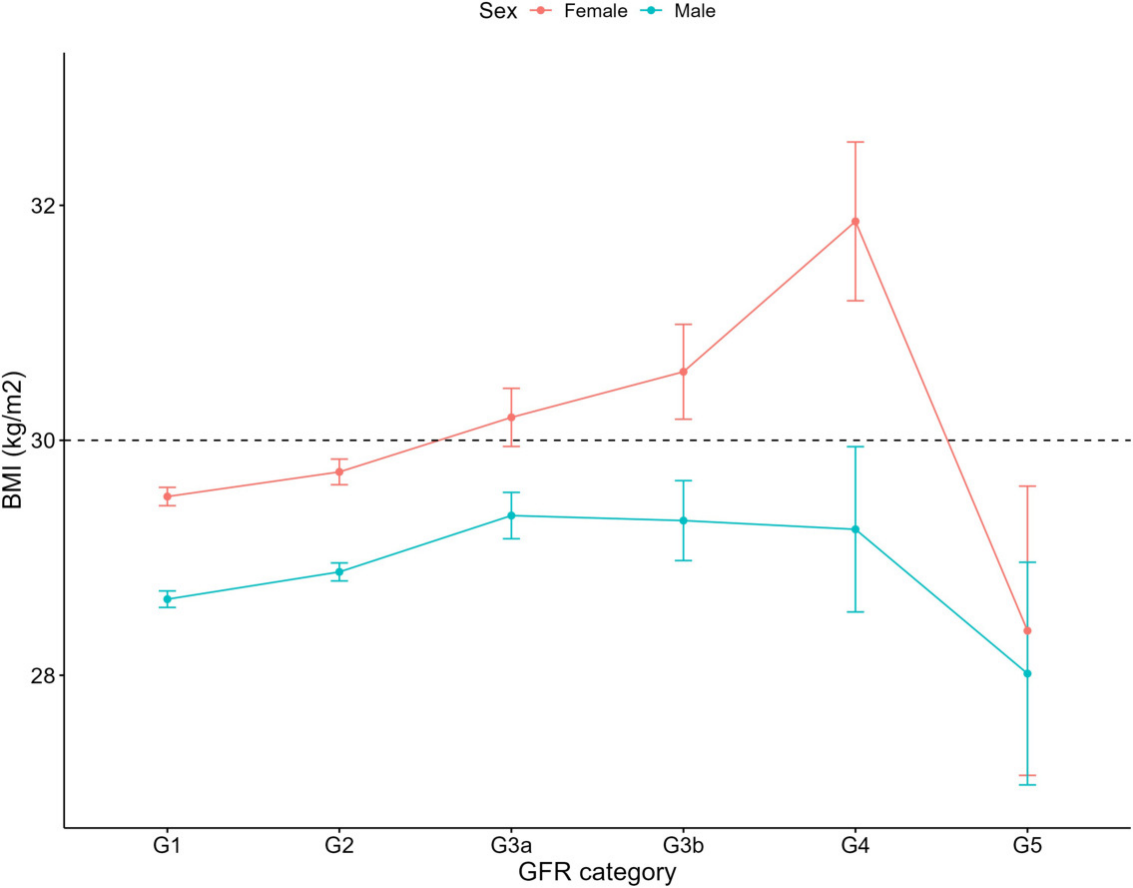
Body mass index within each sex by GFR category, NHANES (2007–2018). Figure illustrates body mass index (BMI; kg/m^2^) for females and males, grouped by GFR category. Dashed line indicates the cut-off value used in obese classification (30 kg/m^2^).

**Figure 4:**
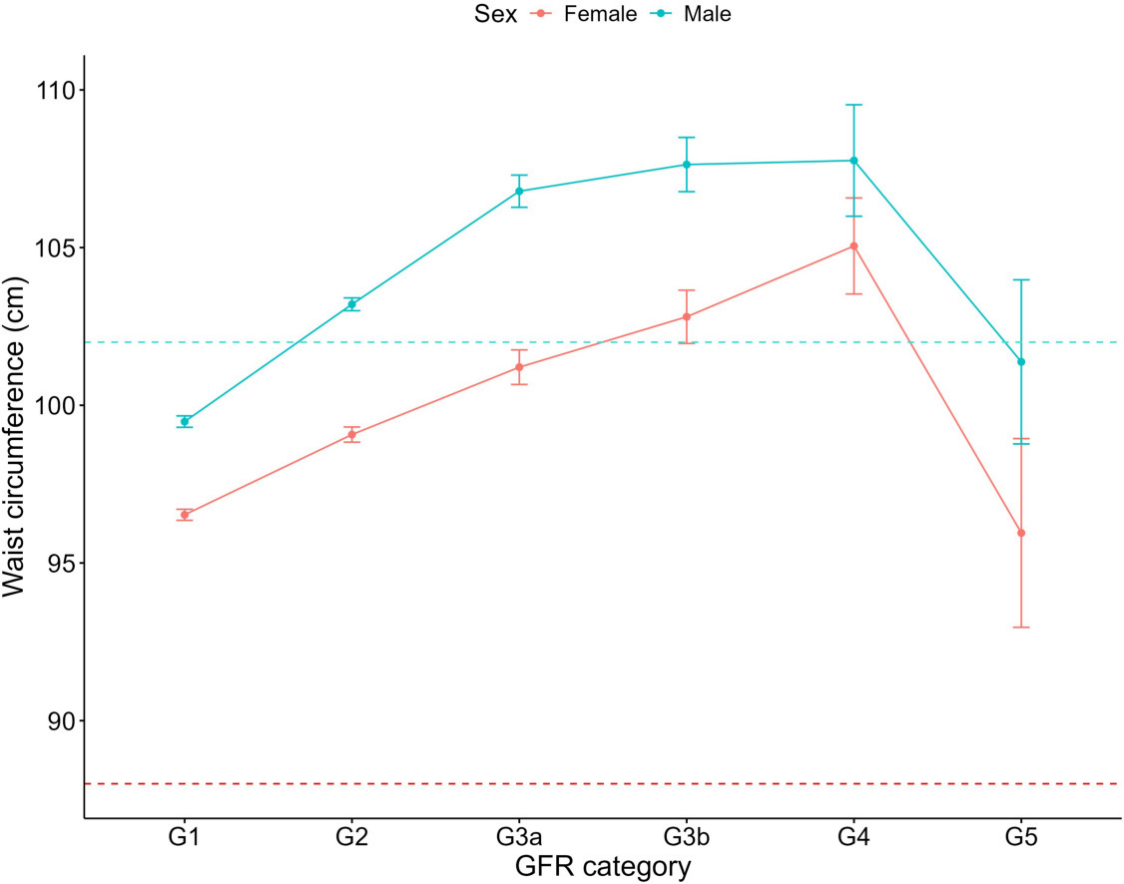
Waist circumference within each sex by GFR category, NHANES (2007–2018). Figure illustrates waist circumference (cm) for females and males, grouped by GFR category. Dashed red line indicates the cut-off value used in high waist circumference classification for females (88 cm) and dashed blue line indicates the cut-off value used for males (102 cm).

**Figure 5:**
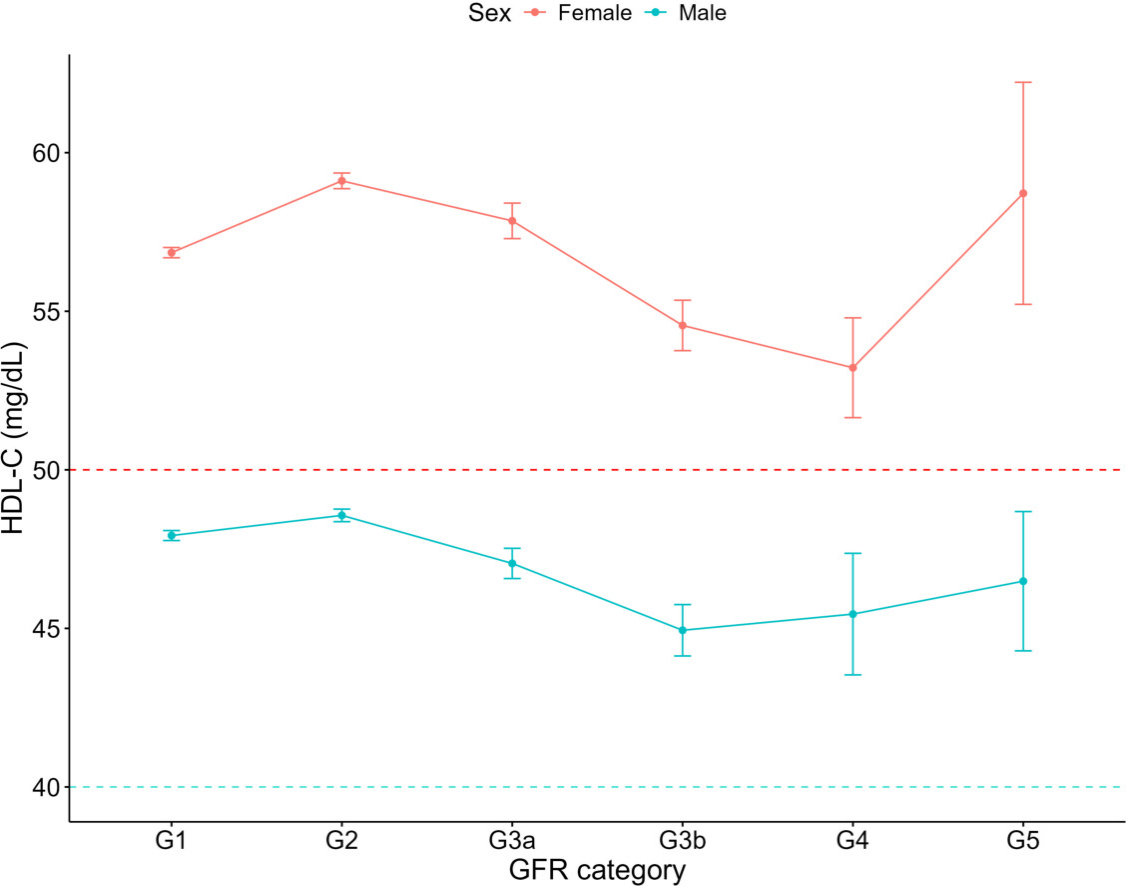
HDL-C within each sex by GFR category, NHANES (2007–2018). Figure illustrates HDL-C (mg/dl) for females and males, grouped by GFR category. Dashed red line indicates the cut-off value used for low HDL-C classification for females (50 mg/dl) and dashed blue line indicates the cut-off value used for males (40 mg/dl).

Increasing systolic blood pressure with GFR category was observed for both females and males, with a notable increase above the defined hypertension threshold after G4 (Figure 1). Conversely, diastolic blood pressure did not show a clear relationship with GFR categories (Figure 2). As detailed in Table 3, overall, females had significantly higher mean BMI values compared to males. BMI in females with CKD showed a transient increase above the cut off in category G4 (relative to G1 and G5), while there was no significant GFR category effect for males (Figure 3). There was a significant increase in waist circumference in males with CKD between G1 to G3a, increasing above the cut-off point (Figure 4). In females, there was an increase between G2 and G4, and a decrease in waist circumference at G5 relative to G4. There were no significant changes in HDL-C throughout GFR category (Figure 5), however, females overall exhibited significantly higher mean levels of HDL-C (Table 3).

**Table 3:** Mean values of cardiovascular risk factors for females and males with and without CKD, NHANES (2007–2018).

Variables	Mean (±SD)		*p*-value
	Female	Male	
Systolic blood pressure (mmHg)	122.8 ± 19.8	125.9 ± 17.1	<0.05
Diastolic blood pressure (mmHg)	68.8 ± 12.6	71.7 ± 12.9	<0.05
BMI (kg/m^2^)	29.7 ± 7.7	28.7 ± 6.1	<0.05
HDL-C (mg/dl)	57.4 ± 16.4	48.0 ± 14.5	<0.05
Waist circumference (cm)	97.7 ± 16.7	101.3 ± 15.8	<0.05
Haemoglobin (g/dl)	13.2 ± 1.3	14.9 ± 1.3	<0.05
Haematocrit (%)	39.1 ± 3.5	43.7 ± 3.7	<0.05
Triglycerides (mg/dl)	138.7 ± 106.6	170.6 ± 149.3	<0.05
Albumin (g/dl)	4.1 ± 0.3	4.3 ± 0.3	<0.05

*p*-value indicates the statistical differences between females and males.

**Figure 6:**
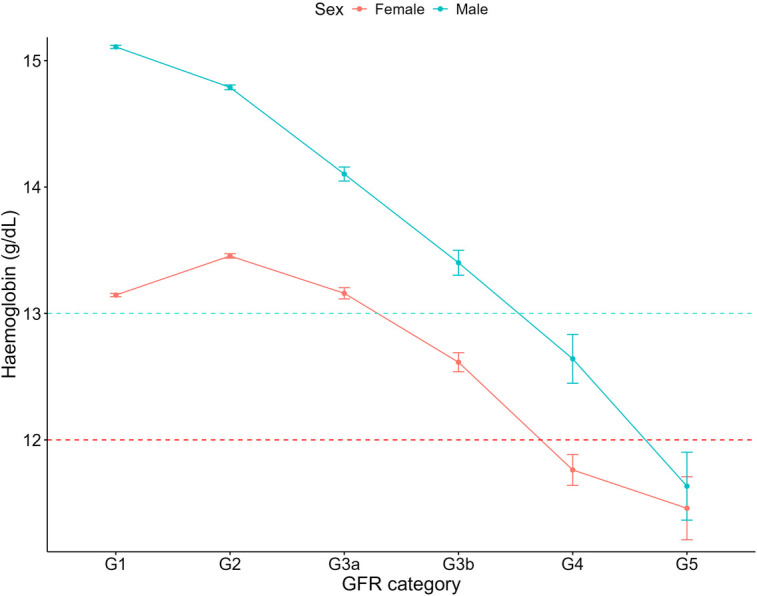
Haemoglobin within each sex by GFR category, NHANES (2007–2018). Figure illustrates haemoglobin (g/dl) for females and males, grouped by GFR category. Dashed red line indicates the cut-off value used for a low haemoglobin classification in females (12 g/dl) and the dashed blue line indicates the cut-off value used for males (13 g/dl).

**Figure 7:**
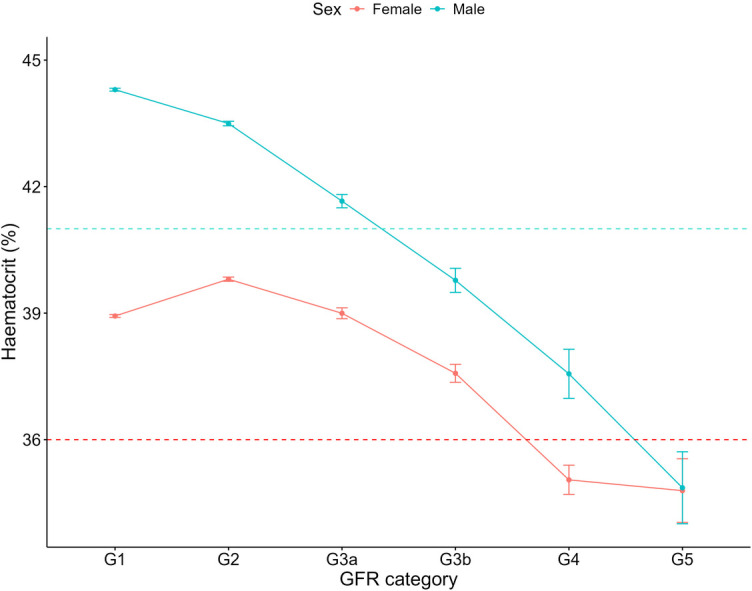
Haematocrit within each sex by GFR category, NHANES (2007–2018). Figure illustrates haematocrit (%) for females and males, grouped by GFR category. Dashed red line indicates the cut-off value used in low haematocrit classification for females (36%) and dashed blue line indicates the cut-off value used for males (41%).

**Figure 8:**
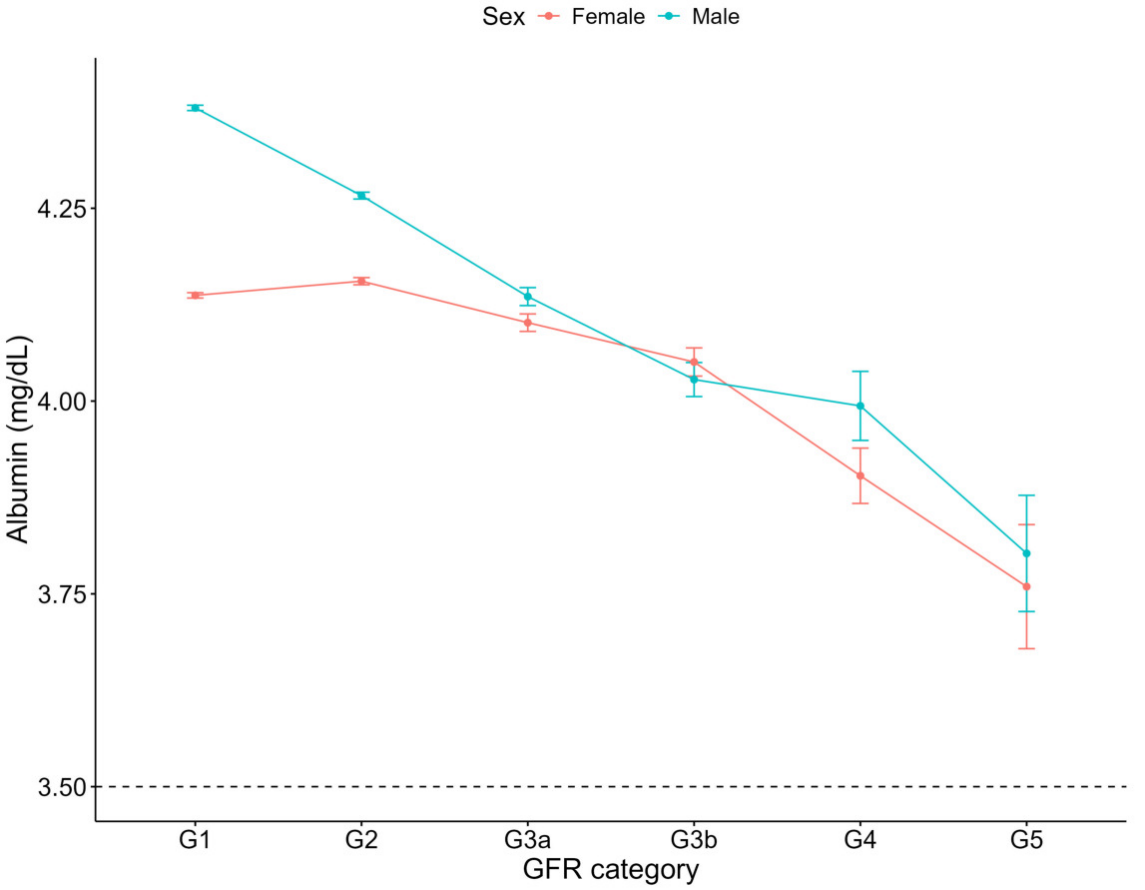
Albumin within each sex by GFR category, NHANES (2007–2018). Figure illustrates albumin (mg/dl) for females and males, grouped by GFR category. Dashed line indicates the cut-off value used in low albumin (3.5 mg/dl).

**Figure 9:**
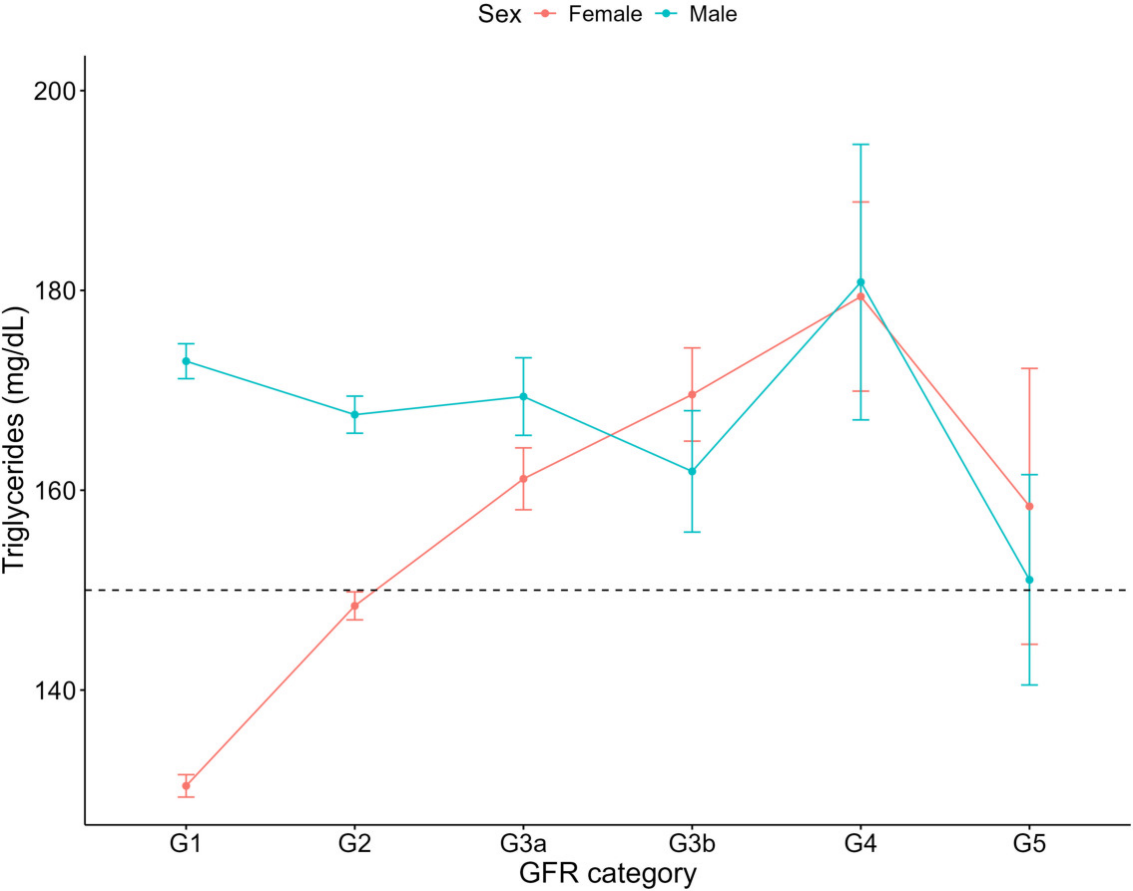
Triglycerides within each sex by GFR category, NHANES (2007–2018). Figure illustrates triglycerides (mg/dl) for females and males, grouped by GFR category. Dashed line indicates the cut-off value used in elevated triglycerides (150 mg/dl).

For both haemoglobin and haematocrit, males overall exhibited higher levels compared to females (Table 3). Haemoglobin and haematocrit declined across all GFR categories for males with CKD (Figures 6, 7). For females with CKD, a decline was evident between G2-G4. At G5, haemoglobin and haematocrit levels were the same in males and females. Albumin levels were shown to decrease as renal function declined in both sexes (Figure 8). Triglycerides significantly increased between G1 and G4 for the females, while in the males, there was no significant difference in triglycerides between GFR categories (Figure 9).

### 3.3 Assessing sex differences in cardiovascular risk factors among those with and without CKD

Females with CKD demonstrated 46% reduced odds of diabetes (OR: 0.54, CI: 0.43–0.67) and 19% reduced odds of hypertension (OR: 0.81, CI: 0.67–0.98), compared to males with CKD (Table 4). Females with CKD also exhibited reduced odds for low haematocrit (OR: 0.48, CI: 0.40–0.75) and elevated triglycerides (OR: 0.75, CI: 0.63–0.88) compared to males with CKD. Females without CKD exhibited lower odds of diabetes (OR: 0.80, CI: 0.69–0.94), hypertension (OR: 0.85, CI: 0.79–0.91), low haematocrit (OR: 0.86, CI: 0.78–0.96), and elevated triglycerides (OR: 0.55, CI: 0.50–0.60), compared to males without CKD.

**Table 4:** Multivariable logistic regression of the association between sex and various cardiovascular risk factors in individuals with and without CKD in the sample, NHANES (2007–2018).

Variables	Individuals with CKD		Individuals without CKD	
	Adjusted OR (95% CI)	*p*-value	Adjusted OR (95% CI)	*p*-value
Diabetes				
Female (vs. male)	0.54 (0.43–0.67)	<0.05	0.80 (0.69–0.94)	<0.05
Hypertension				
Female (vs. male)	0.81 (0.67–0.98)	<0.05	0.85 (0.79–0.91)	<0.05
Obesity				
Female (vs. male)	0.87 (0.74–1.02)	0.08	1.03 (0.95–1.12)	0.51
High waist circumference				
Female (vs. male)	1.69 (1.40–2.04)	<0.05	2.32 (2.16–2.51)	<0.05
Low HDL-C				
Female (vs. male)	1.18 (1.00–1.39)	0.05	1.11 (1.02–1.20)	<0.05
Low haemoglobin				
Female (vs. male)	0.98 (0.78–1.22)	0.85	2.70 (2.23–3.28)	<0.05
Low haematocrit				
Female (vs. male)	0.48 (0.40–0.57)	<0.05	0.86 (0.78–0.96)	<0.05
Low albumin				
Female (vs. male)	1.10 (0.73–1.64)	0.65	5.18 (3.70–7.25)	<0.05
Elevated triglycerides				
Female (vs. male)	0.75 (0.63–0.88)	<0.05	0.55 (0.50–0.60)	<0.05

*p*-value indicates statistical significance for each multivariable logistic regression.

Females both with and without CKD exhibited substantially increased odds of a high waist circumference (OR: 1.69, CI: 1.40–2.04 and OR: 2.32, CI: 2.16–2.51, respectively), and low HDL-C (OR: 1.18, CI: 1.00–1.39 and OR: 1.11, CI: 1.02–1.20, respectively) compared to their male counterparts. Additionally, females without CKD exhibited increased odds of having low haemoglobin (OR: 2.70, CI: 2.23–3.28), and hypoalbuminemia (OR: 5.18, CI: 3.70–7.25), compared to males without CKD.

## 4 Discussion

Our findings reinforce the notion that sex-based disparities in cardiovascular risk are present in individuals with CKD, specifically in how sex affects cardiovascular risk factors and their association with declining renal function. For example, females with CKD exhibited lower odds of diabetes, hypertension, and abnormal lipid profiles compared to males with CKD, yet had higher odds of a high waist circumference and low HDL-C. These results highlight the need for tailored approaches in managing cardiovascular risk in individuals with CKD, considering the distinct profiles of females and males.

Our findings revealed a progressive elevation in systolic blood pressure with advancing CKD in both sexes. This is significant given the association between elevated systolic blood pressure and increased cardiovascular risk (31). For example, a Hong-Kong based retrospective cohort study showed that a 10 mmHg increase in systolic blood pressure is associated with a 11% higher risk of cardiovascular disease (32), and in individuals with CKD, prospective longitudinal studies have shown that a 5 mmHg increase in blood pressure raises the risk of left ventricular hypertrophy by 3% (33). Our data reveal that hypertension affects 71.3% of individuals with CKD, in stark contrast to 33.1% in those without CKD. These results underscore the critical importance of targeted blood pressure management strategies in those with CKD.

Notably, females with and without CKD exhibited odds of hypertension that were 19 and 15 times lower, respectively, than those of their male counterparts. This does align with the literature showing females are at an overall reduced risk of hypertension compared to males (31), though this is impacted by factors such as age (34). While there is less substantive evidence in the CKD population, given the strong links between hypertension and cardiovascular mortality, it does suggest that for hypertension, males are at increased risk (35). This relative reduced risk in females has been attributed to various factors such as sex hormones and renin–angiotensin–aldosterone system mediators, as well as socioeconomic factors like access to health care and adherence to medications (36).

Our analysis revealed that overall, females had higher mean BMI levels than males. However, BMI levels may not be particularly relevant for individuals with kidney disease. Research has indicated an “obesity paradox”, where a higher BMI is associated with better cardiovascular outcomes in those with renal failure (37). Instead, waist circumference and waist-to-hip ratio are considered better indicators of cardiovascular risk (38). Interestingly, our analysis of both BMI and waist circumference by sex and GFR category, revealed a decline in both measures for females at G5 (relative to G4) but not for males. This could be attributed to malnutrition or frailty which is commonly observed in those with CKD, arising from numerous factors including the uremic state, protein energy wasting and reduced food intake (39). Why however, there is a difference between females and males is not clear and warrants further investigation.

Moreover, in our study, while males had an overall higher average waist circumference, females significantly exceeded the high-risk threshold (88 cm vs. 102 cm for males). In a BMI-adjusted model, Postorino et al. revealed that waist circumference was a direct predictor of cardiovascular mortality (40). The strong link between waist circumference and adverse health outcomes suggests an elevated risk for females with CKD, with our data showing they were 1.69 times more likely to have a high waist circumference than their male counterparts, and females without CKD who were 2.32 times more likely to have a high waist circumference. Despite strong evidence that waist circumference provides crucial insights beyond BMI for predicting health outcomes, it is underutilized in clinical settings, representing a missed opportunity for enhanced cardiovascular risk assessment, specifically for females who may face a heightened cardiovascular risk compared to their male counterparts (41).

Our analysis identified a decline in HDL-C between GFR categories G2 and G4, for both sexes and that females with and without CKD showed marginally increased odds of low HDL-C compared to males. Lower HDL-C levels have been considered a risk factor for cardiovascular disease (42), however interventions targeting HDL-C elevation have not reduced cardiovascular events in patients (43). Indeed, higher HDL-C levels have been associated with accelerated GFR decline in middle-aged populations without pre-existing conditions (44). This may be linked to the functionality of HDL particles, whereby inflammatory enzymes, hyperglycaemia and oxidative stress may cause dysfunctional remodelling of the HDL-C particles (44). As such, while HDL-C dysfunction contributes to cardiovascular risk in CKD, therapeutic strategies targeting HDL-C metabolism require further investigation, which will need to consider the effect of sex.

Anaemia, characterized by a reduction in haemoglobin, haematocrit or red blood cell count, is common in kidney disease and typically worsens with disease progression (45, 46). Consistent with this, our data revealed a decline in haemoglobin and haematocrit levels with advancing CKD. Overall, males had significantly higher mean levels of both haemoglobin and haematocrit compared to females. Among individuals without CKD, females showed over double the odds of low haemoglobin compared to males, aligning with previous studies (47). However, our analysis of haemoglobin and haematocrit by sex and GFR category revealed that in patients with renal failure (G5), haemoglobin and haematocrit levels were similar between the sexes. This is likely due to decreased erythropoietin production from reduced renal function (45). Our results suggest that while females without CKD are at greater odds of low haemoglobin, the male advantage diminishes in the context of CKD, as both sexes face a similar likelihood of anaemia. This compares with the KNOW-CKD Korean prospective cohort study, where female patients demonstrated a higher anaemia prevalence in early CKD stages (1–3b) but this sex-based difference disappeared in advanced stages (4–5) (48). We have shown previously that females and males with CKD may have a relatively equal risk of cardiovascular mortality (12), and this data suggest that anaemia as a cardiovascular risk factor, may carry comparable risk for both females and males in advanced CKD. Future research into the impact of anaemia on cardiovascular outcomes in CKD could provide valuable insights and inform more effective management strategies.

Our analysis revealed a decline in albumin as renal function declined, which was evident in both sexes, although the mean albumin levels did not drop below the cutoff value of 3.5 g/dl, which is commonly used to define hypoalbuminemia (26). Low serum albumin is linked to increased risk and poorer outcomes in cardiovascular disease, including heart failure, stroke, and coronary artery disease, independent of traditional risk factors (49). It is a potentially modifiable risk factor, with interventions to raise albumin levels a possible approach for patients with CKD. For example, in another United States based population study, Leon et al. (50) conducted a randomized controlled trial to assess whether addressing nutritional barriers could enhance albumin levels in hemodialysis patients. Their findings demonstrated that personalized nutrition interventions led to improved serum albumin levels among patients with limited nutritional knowledge.

Of note is that in our study, among individuals with CKD, low serum albumin levels did not significantly differ between females and males. Albuminuria, a key marker of kidney damage and progression and is a defining feature of CKD (16). The pathophysiological processes associated with albuminuria include glomerular dysfunction and tubular damage, and are closely associated with endothelial injury and podocyte loss (51). This widespread pathological feature driving persistent protein loss into the urine may obscure potential sex-based differences in albumin metabolism. In contrast, among individuals without CKD, our study identified a significant sex difference, with females exhibiting 5.18 times increased odds of hypoalbuminemia compared to males. Serum albumin levels have been found to decrease in females post-puberty more rapidly than males (52, 53), which may explain our findings.

Our analysis found that females with and without CKD had 50% and 20% lower odds of diabetes compared to males, respectively. Our data thus shows that males face a greater diabetes burden, both in the presence and absence of CKD, with the disparity being slightly more pronounced among those with CKD. This aligns with other studies such as Nordstrom et al.'s Swedish based prospective population study of older age individuals, which found that males were at almost double the risk of type 2 diabetes than older females (54). However, findings on the relationship between sex, diabetes and CKD are not consistent across all studies. For example, Yang et al's large-scale cross-sectional study in Shanghai reported that females with CKD were more likely to have diabetes than males (55). There is a complex interrelationship with diabetes and CKD, with diabetic kidney disease being the primary cause of CKD (56). Why diabetes and CKD present differently in females and males has been attributed to variations in sex hormone signaling, renal glucose handling, and gene expression, and how these factors influence susceptibility to hyperglycemia, glomerular hyperfiltration, vascular health and disease progression (57). Our data underscore the importance of considering sex differences in the management of diabetes as a cardiovascular risk factor in individuals with CKD. Future research should investigate how sex-specific factors influence glycemic control and the efficacy of diabetes treatments, such as SGLT2 inhibitors and GLP-1 receptor agonists, which have demonstrated cardiorenal benefits (58). Additionally, exploring disparities in glycemic and cardiovascular risk management including blood pressure control between sexes is crucial, as women with diabetes are often undertreated (59). This will allow personalized strategies for both impacting modifiable risk factors and improving outcomes in both females and males with CKD and diabetes (59).

The NHANES database is robust due to its large sample size of over 30,000 participants and its use of weightings to address oversampling and bias and to ensure representativeness. It benefits from objective measurements and for our purposes, a reduction in selection bias as the sample isn't specifically chosen for CKD assessment. However, there are notable limitations. The reliance on self-reported data introduces potential recall and response biases, which may affect accuracy. Further, the NHANES uses a single measurement for a variety of biomarkers, including those used for assessing renal function. This method differs from KDIGO's 2024 guidelines which recommend measurements over a 3-month period (16). This could lead to misclassifying episodes of acute kidney injury as CKD. Additionally, the cross-sectional nature of the NHANES introduces limitations in the study, such as the inability to determine cause-and-effect relationships (60). Moreover, while we accounted for ethnicity in our analyses, we did not explore potential interactions between sex and ethnicity in relation to cardiovascular risk factors. Given the potential impact of these interactions on cardiovascular outcomes, future research should investigate how sex and ethnicity together may contribute to disparities in cardiovascular health.

In conclusion, this cross-sectional study reveals sex-based differences in cardiovascular risk factors in individuals with CKD and further that many of these risk factors change significantly in their presentation across the course of disease progression in both sexes. Using this more nuanced understanding of the variability of these risk factors is crucial for mitigating their impact on cardiovascular mortality in females with CKD, especially considering that the effects of CKD on females have often been underestimated to date (4). This work adds to the body of evidence that challenges the conventional understanding of sex disparities in cardiovascular risk and emphasize the importance of integrating sex-specific perspectives into both CKD research and management to enhance personalized care.

## Data Availability

Publicly available datasets were analyzed in this study. This data can be found here: https://wwwn.cdc.gov/nchs/nhanes/Default.aspx.
